# Interplay of Proximal Flow Confluence and Distal Flow Divergence in Patient-Specific Vertebrobasilar System

**DOI:** 10.1371/journal.pone.0159836

**Published:** 2016-07-28

**Authors:** Xiaoping Yin, Xu Huang, Yundi Feng, Wenchang Tan, Huaijun Liu, Yunlong Huo

**Affiliations:** 1 Department of Medical Imaging, The Second Hospital of Hebei Medical University, Shijiazhuang, China; 2 Department of Mechanics and Engineering Science, College of Engineering, Peking University, Beijing, China; 3 State Key Laboratory for Turbulence and Complex Systems, College of Engineering, Peking University, Beijing, China; 4 Shenzhen Graduate School, Peking University, Shenzhen, China; 5 College of Medicine, Hebei University, Baoding, China; Technion—Israel Institute of Technology, ISRAEL

## Abstract

Approximately one-quarter of ischemic strokes involve the vertebrobasilar arterial system that includes the upstream flow confluence and downstream flow divergence. A patient-specific hemodynamic analysis is needed to understand the posterior circulation. The objective of this study is to determine the distribution of hemodynamic parameters in the vertebrobasilar system, based on computer tomography angiography images. Here, the interplay of upstream flow confluence and downstream flow divergence was hypothesized to be a determinant factor for the hemodynamic distribution in the vertebrobasilar system. A computational fluid dynamics model was used to compute the flow fields in patient-specific vertebrobasilar models (n = 6). The inlet and outlet boundary conditions were the aortic pressure waveform and flow resistances, respectively. A 50% reduction of total outlet area was found to induce a ten-fold increase in surface area ratio of low time-averaged wall shear stress (i.e., TAWSS ≤ 4 dynes/cm^2^). This study enhances our understanding of the posterior circulation associated with the incidence of atherosclerotic plaques.

## Nomenclature

1. **SAR-TAWSS at a junction**: surface area ratio of low TAWSS ($=\frac{{\text{Surface area}}_{\text{TAWSS}\leq {4\;\text{dynes}/\text{cm}}^{2}}}{\text{Junctional surface area}}\times 100\%$). “Junctional surface area” refers to the total surface area of mother and daughter vessels of 1cm length.
2. **SAR-TAWSS in a vessel**: surface area ratio of low TAWSS ($=\frac{{\text{Surface area}}_{\text{TAWSS}\leq {4\;\text{dynes}/\text{cm}}^{2}}}{\text{Vessel surface area}}\times 100\%$). “Vessel surface area” refers to the total surface area of a vessel.
3. **SAR-OSI at a junction**: surface area ratio of high OSI ($=\frac{{\text{Surface area}}_{\text{OSI}\geq 0.15}}{\text{Junctional surface area}}\times 100\%$).
4. **SAR-OSI in a vessel**: surface area ratio of high OSI ($=\frac{{\text{Surface area}}_{\text{OSI}\geq 0.15}}{\text{Vessel surface area}}\times 100\%$).
5. **SAR-transWSS at a junction**: surface area ratio of high transWSS ($=\frac{{\text{Surface area}}_{\text{transWSS}\geq {6\;\text{dynes}/\text{cm}}^{2}}}{\text{Junctional surface area}}\times 100\%$).
6. **SAR-transWSS in a vessel**: surface area ratio of high transWSS ($=\frac{{\text{Surface area}}_{\text{transWSS}\geq {6\;\text{dynes}/\text{cm}}^{2}}}{\text{Vessel surface area}}\times 100\%$).

## Introduction

Approximately 80% of strokes are attributed to the ischemic origin, of which 20% to 25% are located in posterior circulation [1]. The posterior circulation is generally supplied by the vertebrobasilar arterial system comprised of left and right vertebral arteries (VA), anterior inferior cerebellar artery (AICA), basilar artery (BA), left and right superior cerebellar arteries (SCA), and left and right posterior cerebral arteries (PCA) [2]. The most common sites of atherosclerotic diseases, in order, are the BA, VA and PCA in the vertebrobasilar system [3, 4].

Abnormal hemodynamic parameters (e.g., low wall shear stress, WSS; high oscillatory shear index, OSI; and high transverse WSS, transWSS) have been found to contribute to the incidence and progression of atherosclerosis [5–8]. The BA is the only large artery with converging flow patterns from left and right VAs in the cardiovascular system. Computational fluid dynamic (CFD) methods have been used to extensively investigate the distribution of hemodynamic parameters near the anastomosis of BA and VAs [9–14]. There is, however, lack of patient-specific hemodynamic studies in the vertebrobasilar system including VAs, AICA, BA, SCAs and PCAs.

The objective of this study is to carry out a hemodynamic analysis in the vertebrobasilar system, based on patient computer tomography angiography (CTA) images. Here, we hypothesized that flow patterns in BA are mainly determined by the interplay of upstream flow confluence (LVA and RVA merging into BA) and downstream flow divergence (BA bifurcating into SCAs and PCAs). To test the hypothesis, a transient 3D finite volume model was used to solve continuity and Navier-Stokes equations to compute the flow field based on the reconstructed geometry from CTA images. The inlet (at the inlet of LVA and RVA) and outlet boundary conditions (at the outlet of AICA, SCAs and PCAs) were the aortic pressure wave and flow resistances, respectively. Hemodynamic parameters including TAWSS (time-averaged WSS over a cardiac cycle), OSI and transWSS were computed from the flow field. The significance, implication and limitation were discussed in relation to the incidence of atherosclerosis and aneurysms in the vertebrobasilar system.

## Materials and Methods

### Study design

The purpose of this retrospective study was to investigate the hemodynamic changes in the vertebrobasilar system given a disproportionately high rate of stroke mortality in China [15]. Six patients underwent cerebral CTA at the affiliated hospital of Hebei University, China, to evaluate impairments in vision, body movement, and speaking; unconsciousness; problems with coordination and so on. Table 1 summarizes patient demographics. CTA reconstruction and imaging analysis were performed by researchers at Peking University and Radiologists at the affiliated hospital of Hebei University, which showed no stenoses and aneurysms in patient vertebrobasilar system. This retrospective study was approved by the Institutional Review Board (IRB) for the affiliated hospital of Hebei University. Subjects gave the signed informed consent.

**Table 1:** Demographics of the study population.

Subjects	A	B	C	D	E	F
Age (y)	58	65	59	42	55	51
Gender	Male	Male	Male	Female	Male	Male
BMI* (kg/m^2^)	22.86	29.04	27.36	22.4	26.87	19.96
Blood pressure (mmHg)						
Systolic	124	153	128	130	135	145
Diastolic	85	81	85	80	78	89
Hypertension	N	Y	N	N	N	Y
Diabetes mellitus	N	N	N	N	N	N
Active smoker	Y	Y	Y	N	Y	Y
Family history of CAD*	N	unknown	unknown	unknown	N	N
Total cholesterol (mmol/L)	3.81	5.9	3.2	3.65	3.69	3.11
Triglycerides (mmol/L)	1.68	3.04	0.99	1.88	1.62	0.52
LDL* (mmol/L)	2.18	3.74	1.95	2.05	2.3	1.68
HDL* (mmol/L)	0.79	0.8	0.92	0.67	0.72	0.95
Fasting glucose (mmol/L)	4.43	4.45	5.03	4.43	5.32	4.62

*BMI = Body mass index, CAD = Coronary artery disease, LDL = Low density lipoprotein, HDL = High density lipoprotein

### Imaging acquisition

Similar to a previous study [16], all patients underwent CTA scanning from the aortic arch to vertex using the Discovery CT750 HD scanner (HDCT, GE Healthcare, Milwaukee, WI, USA). Briefly, non-enhanced CT brain scan was first performed, which was followed by contrast enhanced CTA. CTA images were acquired when contrast agent (Iopromide 370, Bayer Schering Pharma AG) at the dose of 1.0 ml/kg was injected at a rate of 5 ml/s followed by IV injection of saline chase of 40 ml at a rate of 5 ml/s. A bolus tracking method (Smart Prep) was used to monitor the optimal contrast enhancement. Study parameters included the caudo-cranial scan direction, 120 kVp, rotation time of 0.5 s, 50cm × 50cm DFOV (Display Field Of View), 0.625 mm construction thickness at 0.625 mm intervals, and helical pitch of 0.984:1.

### Aortic pressure wave

A patient with coronary artery diseases underwent the examination of invasive coronary angiogram by standard catheterization in accordance with the American College of Cardiology Guidelines for Coronary Angiography [17]. The blood pressure wave was measured by a pressure catheter inserted into the ascending aorta, which was monitored by a pressure control unit (Millar INC, Houston).

### 3D reconstruction

**Fig 1:**
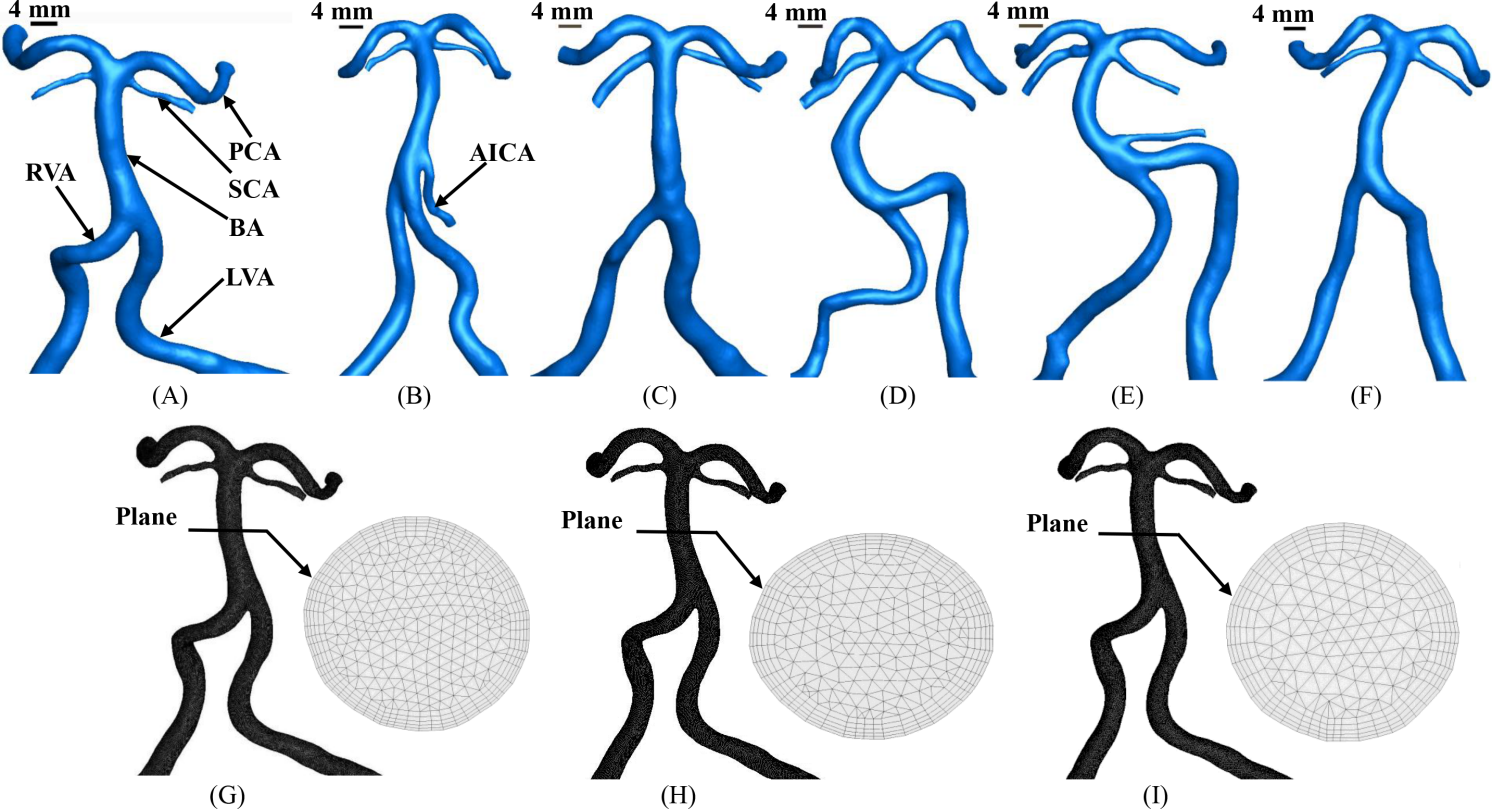
(A-F) 3D geometry of vertebrobasilar arteries reconstructed from CTA images of human subjects A-F, respectively; (G-I) meshes of vertebrobasilar arteries of human subject A, where (G) maximal element size = 0.25 mm, (H) maximal element size = 0.3 mm, (I) maximal element size = 0.35 mm with finer meshes near the wall.

3D geometry and morphometry of vertebrobasilar arteries were extracted from CTA images using the MIMICS software (Materialise, NV, Belgium), as shown in Fig 1A–1F and Table 2. We focused on the intracranial vertebrobasilar system that is comprised of VAs (LVA and RVA), BA, AICA, SCAs (LSCA and RSCA) and PCAs (LPCA and RPCA). The left and right VAs join at the pontomedullary junction forming the BA which bifurcates into the right and left PCAs as well as right and left SCAs at the pontomesencephalic junction. We excluded some side branches of small diameters that were not observed clearly in the reconstruction (a low CT-threshold of 80 HU). After 3D reconstruction of the vertebrobasilar system, centerlines of the VAs, BA, AICA, SCAs and PCAs were first generated. In the MIMICS software, a centerline was formed by a series of center points which was located in the center on the cross–sectional views of the contour of the 3D vessel. Subsequently, the best fit diameter, D_fit_, was calculated as twice the average radius between the point on the centerline and the contour forming the 3D vessel.

**Table 2:** Anatomy of the vertebrobasilar system in human subjects A-F.

Human subjects		A	B	C	D	E	F
LVA (mm)	Mean D_fit_	3.5	3.4	4.5	3.8	3.9	4.4
	Arc Length	42.3	41.4	47.0	51.9	62.7	53.6
	Chord length	36.3	36.3	44.5	36.1	47.5	49.4
RVA (mm)	Mean D_fit_	3.7	3.4	3.3	2.8	3.3	3.7
	Arc Length	46.7	41.1	45.1	35.8	56.6	57.1
	Chord length	39.9	37.2	42.5	22.9	50.4	51.2
BA (mm)	Mean D_fit_	4.3	3.9	4.3	4.3	4.0	4.3
	Arc Length	21.2	25.6	25.9	27.1	24.5	24.8
	Chord length	20.3	23.6	25.2	22.3	19.5	23.3
LSCA (mm)	Outlet D_fit_	1.0	1.2	1.2	2.0	1.5	1.1
	Mean D_fit_	1.4	1.5	1.6	2.2	1.5	1.5
	Arc Length	14.7	13.6	17.2	16.8	20.7	13.5
	Chord length	13.8	12.1	16.6	13.8	18.0	11.8
RSCA (mm)	Outlet D_fit_	0.5	1.0	2.0	2.1	1.8	1.0
	Mean D_fit_	1.3	1.5	2.0	2.3	2.0	1.5
	Arc Length	20.0	10.4	20.4	16.0	21.2	11.3
	Chord length	14.0	9.8	17.3	15.3	18.3	10.5
LPCA (mm)	Outlet D_fit_	2.4	1.7	2.6	2.3	2.2	2.5
	Mean D_fit_	2.7	2.6	2.6	3.0	2.5	2.8
	Arc Length	17.8	21.1	21.6	24.5	20.7	17.0
	Chord length	13.7	15.4	16.7	19.0	17.8	13.4
RPCA (mm)	Outlet D_fit_	3.1	1.7	2.7	2.6	2.2	3.1
	Mean D_fit_	2.7	2.5	2.9	2.9	2.6	2.8
	Arc Length	22.0	19.6	22.1	21.7	22.3	15.3
	Chord length	15.9	14.2	17.5	13.9	16.1	12.0

Mean D_fit_: D_fit_ averaged along the entire length of a vessel

Arc length: The accumulative length along the centerline from the inlet to outlet of a vessel

Chord length: The straight length from the inlet to outlet of a vessel

LVA and RVA: The intracranial portion of VAs only

LPCA and RPCA: P1 segment only, which origins at the BA termination to the posterior communicating artery (PCOM) within interpeduncular cistern

### Mathematical model

Similar to a previous study [18], CFD simulations were performed to analyze the flow patterns in the vertebrobasilar system. The vessel wall was assumed to be rigid and impermeable. The equations of continuity and Navier-Stokes can be written as:

$$\nabla \cdot \vec{v}=0$$ [1]

$$\rho \frac{\partial \vec{v}}{\partial t}+\rho \vec{v}\cdot \nabla \vec{v}=-\nabla P+\nabla \cdot \mu \left(\nabla \vec{v}+{\left(\nabla \vec{v}\right)}^{T}\right)$$ [2]

where $\vec{v}$, *P*, *ρ*, and *μ* represent the velocity, pressure, blood mass density, and viscosity, respectively. After the flow computation, Reynolds number is determined as: ${\text{Re}}_{\text{mean}}=\frac{\rho {\text{v}}_{mean}D}{\mu }$, where v_*mean*_ and *D* represent the time-averaged velocity and vessel diameter, respectively.

### Method of solution

A finite volume method was applied to solve the governing equations in the ANSYS FLUENT (ANSYS Inc., Canonsburg, USA). Based on these morphometric data, geometrical models were created in the Geomagic Studio software (3D Systems, Rock Hill, USA), which were meshed using the ANSYS ICEM (ANSYS Inc., Canonsburg, USA).

**Fig 2:**
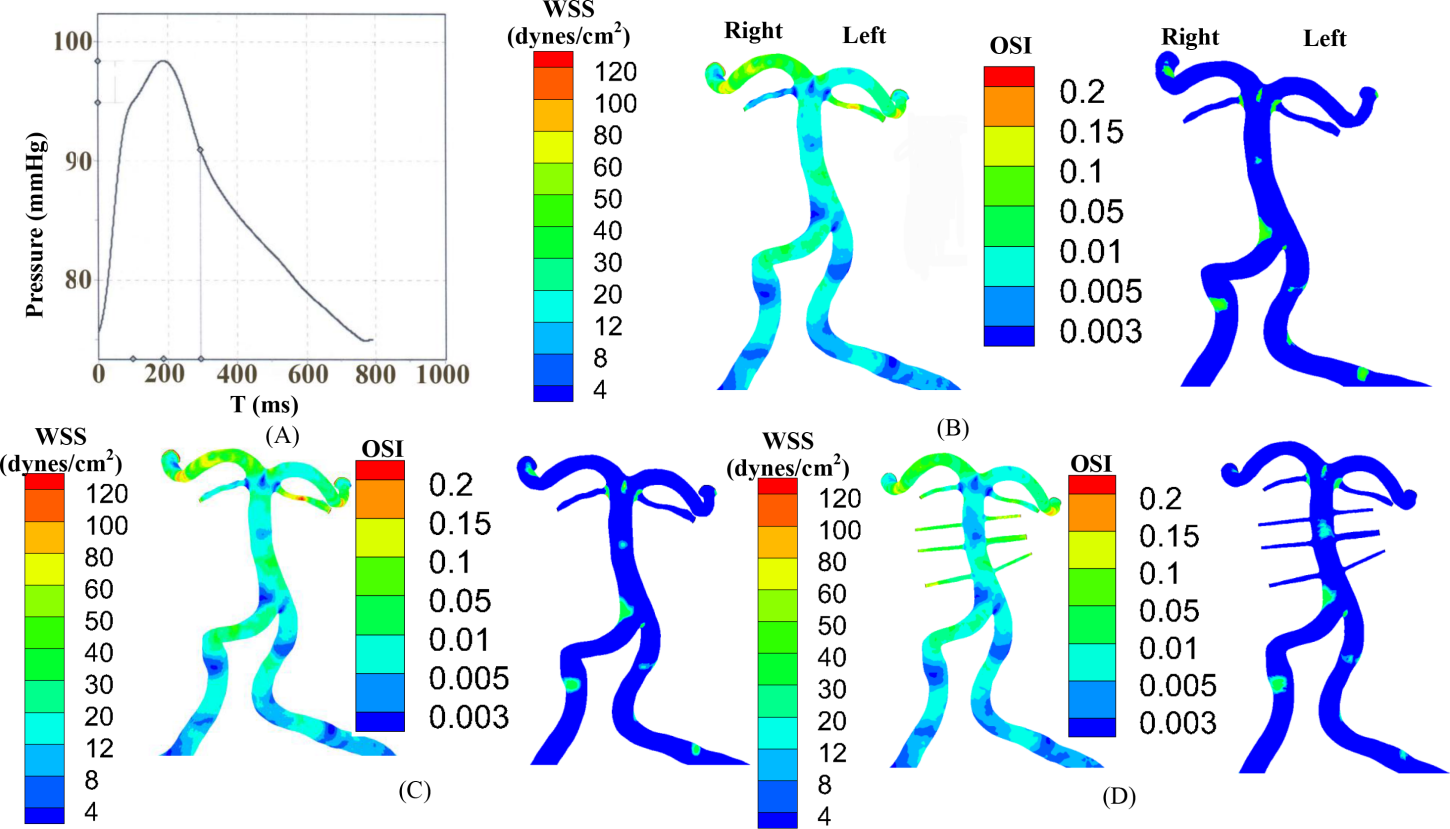
(A) A measured aortic pressure wave; (B-D) TAWSS and OSI in the vertebrobasilar system of human subject A with (B) Newtonian fluid (maximal element size = 0.35 mm), (C) Carreau fluid (maximal element size = 0.3 mm), and (D) Newtonian fluid and artificial small sized branches added to the BA (maximal element size = 0.3 mm).

**Fig 3:**
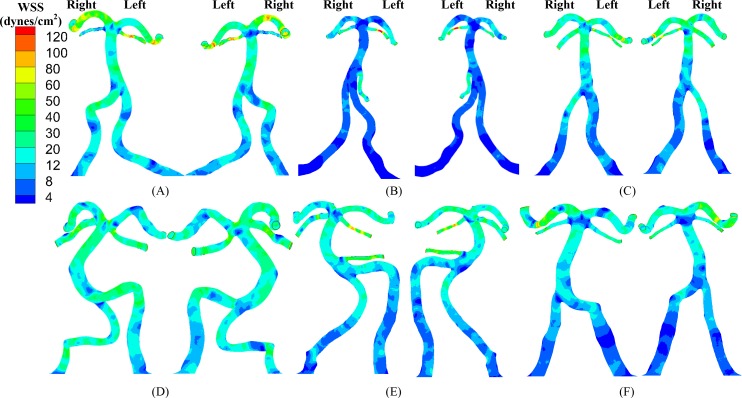
(A-F) TAWSS (left: anterior view; right: posterior view) in the vertebrobasilar system corresponding to Fig 1A–1F.

**Fig 4:**
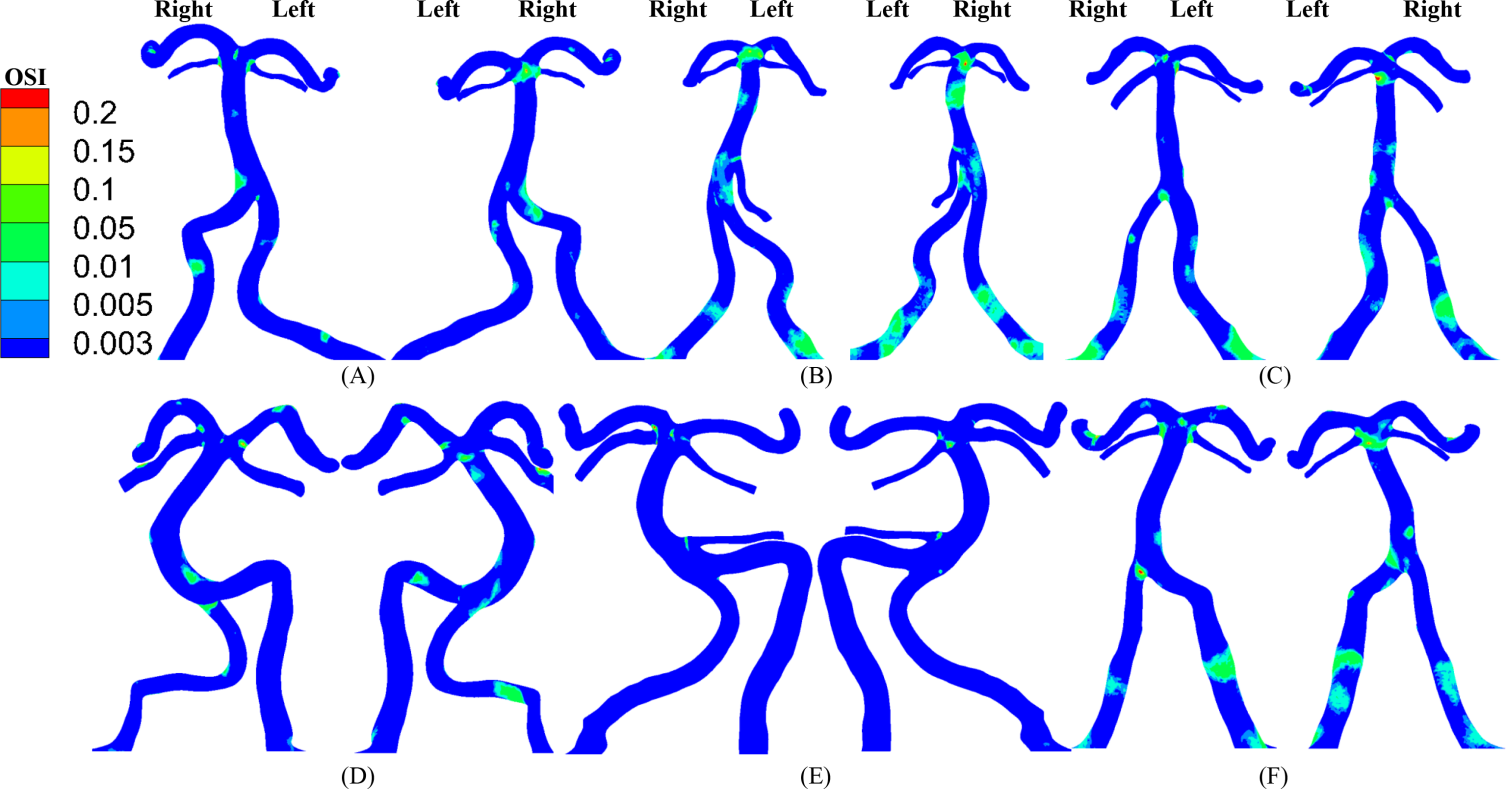
(A-F) OSI (left: anterior view; right: posterior view) in the vertebrobasilar system corresponding to Fig 3A–3F.

Fig 1G–1I show a total of approximately 2.6, 1.8, and 1.3 million hybrid (hexahedral/tetrahedral) shaped volume elements with finer meshes near the vessel wall when the maximal element size was set to 0.35, 0.3, and 0.25 mm, respectively. A mesh dependency was conducted such that the relative error in two consecutive mesh refinements was < 1% for TAWSS and OSI (Fig 2B vs Figs 3A and 4A). A total of approximately 1.8 million hybrid (hexahedral/tetrahedral) shaped volume elements (maximal element size = 0.3 mm) with finer meshes near the vessel wall were necessary to accurately mesh the computational domain.

A measured aortic pressure wave in Fig 2A was applied to the inlet of left and right VAs. The resistance boundary condition was set at each outlet (see the Appendix), based on intraspecific scaling laws of vascular trees that were derived theoretically and validated experimentally in various organs and species [19] including the cerebral circulation [20]. The viscosity and density were set to 4.5×10^−3^ Pa·s and 1,060 kg/m^3^, respectively, to mimic the incompressible blood flow with a hematocrit of 45% [21]. Three cardiac cycles were required to achieve the convergence for the transient analysis.

### Data analysis

The mean±SD (standard deviation) values were computed for TAWSS (averaged over all nodes on the surface of the vertebrobasilar system). Similar to a previous study [18], hemodynamic parameters, i.e., SAR-TAWSS, SAR-OSI, and SAR-transWSS in a vessel and at a junction (see definitions in the nomenclature), were defined to give integral scalar values instead of traditional local parameters. Similar to previous studies [22, 23], a dominant VA was defined as: the diameter of the dominant VA was 0.3 mm larger than another; or the dominant VA straightly connected to the BA if both VAs had similar diameters. ANOVA (SigmaStat 3.5) was used to compare these parameters, where p value < 0.05 represented a statistically significant difference.

## Results

3D geometrical models of the vertebrobasilar system were reconstructed from CTA images of six normal patients (i.e., subjects A-F), as shown in Fig 1A–1F. Accordingly, Table 2 lists mean D_fit_ (averaged along the entire vessel length), arch length and chord length in each artery. At the flow convergence with LVA and RVA merging into BA, subjects A and B have a tuning fork geometry with LVA diameter approximately equal to RVA diameter while subject C has a similar geometry, but a dominant LVA. Subjects D-F have a walking geometry with a dominant LVA. Joining LVA to BA forms an S-shaped LVA-BA in subjects D and E and a C-shaped LVA-BA in subject F. These human subjects have similar junctional shapes of flow divergence that is comprised of BA, LSCA, RSCA, LPCA and RPCA despite various geometrical parameters (e.g., length and diameter).

**Fig 5:**
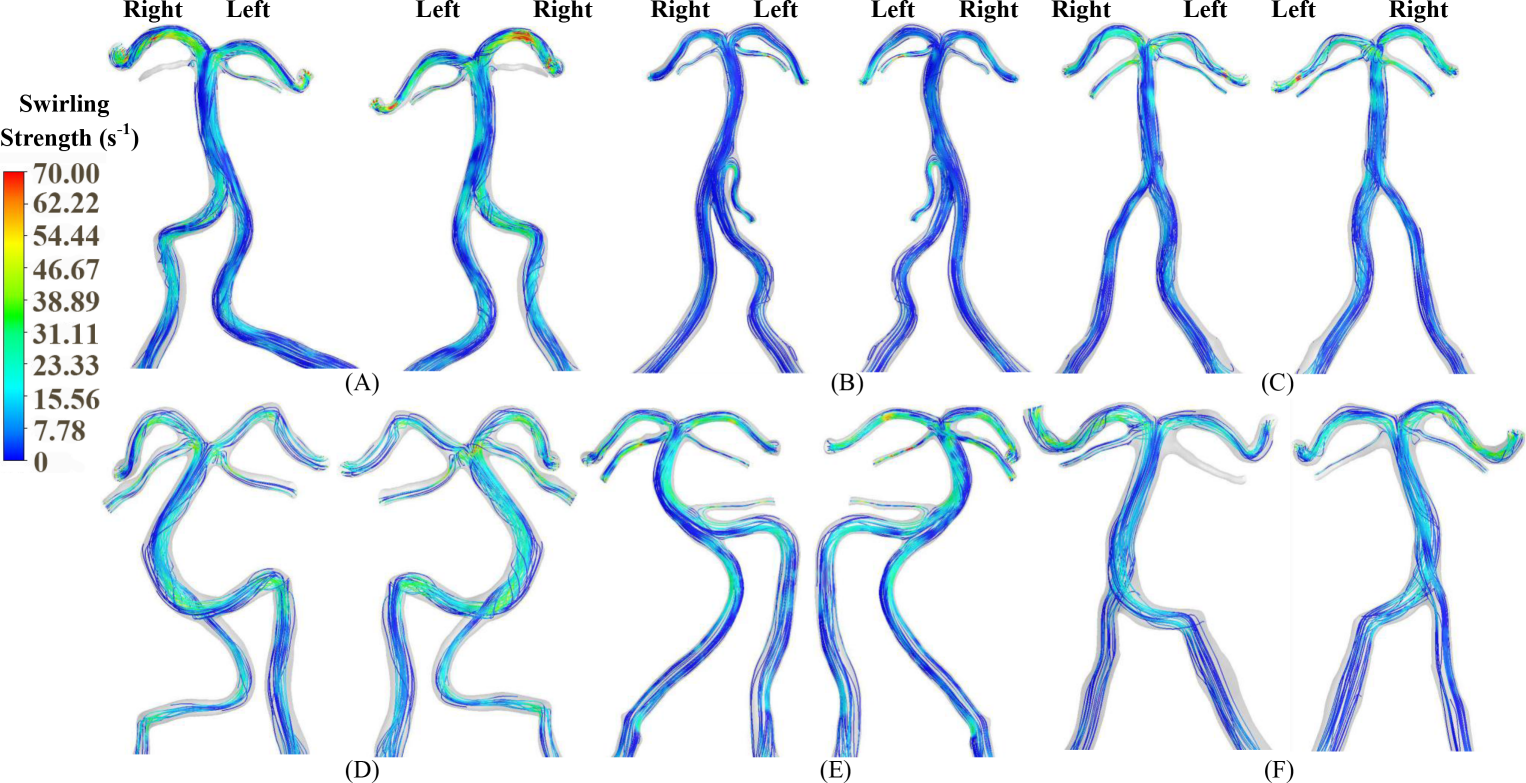
(A-F) Streamlines (left: anterior view; right: posterior view) in the vertebrobasilar system at the time instance with the highest flow velocity at the inlet of VAs (i.e., time equals to 168 ms in Fig 2A) corresponding to Fig 3A–3F.

Fig 2C shows the distribution of TAWSS and OSI in subject A when the blood was considered as a non-Newtonian fluid (i.e., Carreau fluid). Fig 2D shows the two hemodynamic parameters when the blood was considered as a Newtonian fluid as well as artificial small sized branches were added to the BA. Fig 3 shows the distribution of TAWSS, which has mean±SD values of 12.7±12.2, 4.9±6.8, 9.9±8.9, 11.5±8.3, 9.3±8.2, and 7.6±8.1 dynes/cm^2^ in subjects A-F, respectively. Fig 4 shows the corresponding distribution of OSI. A comparison between Fig 2C and 2D and Figs 3A and 4A showed negligible effects of non-Newtonian behavior and possible small sized branches in the BA. There were low TAWSS (≤ 4 dynes/cm^2^) and high OSI (≥ 0.15) near the region of high curvature in each vessel. Table 3 lists SAR-TAWSS and SAR-OSI in each vessel of subjects A-F. Subject B had the maximal SAR-TAWSS in LVA, RVA, BA and LPCA, which were significantly higher than others (p value < 0.05), while subject D had the minimal SAR-TAWSS in those arteries. SAR-OSI had a low value (< 0.3%) and SAR-transWSS was zero in each vessel. Moreover, Fig 5A–5F show the complex distribution of streamlines at the time instance with the highest flow velocity at the inlet of VAs (i.e., time equals to 168 ms in Fig 2A).

**Table 3:** Statistics for SAR-TAWSS and SAR-OSI in each vessel of the vertebrobasilar system of human subjects A-F.

Human subjects		A	B	C	D	E	F
LVA (mm^2^)	Inlet Re_mean_	273	273	281	305	313	361
	Surface area	526	540	645	641	742	634
	SAR-TAWSS (%)	3.5	50.2	8.9	0.8	9.6	19.1
	SAR-OSI (%)	0	0	0	0	0	0
RVA (mm^2^)	Inlet Re_mean_	297	273	281	265	273	386
	Surface area	518	427	461	432	555	449
	SAR-TAWSS (%)	1.6	44.8	26	3.3	9.8	12.8
	SAR-OSI (%)	0	0	0	0	0	0.14
BA (mm^2^)	Surface area	341	414	374	454	338	362
	SAR-TAWSS (%)	2.1	20.3	1.8	0.1	5.7	1.8
	SAR-OSI (%)	0.11	0.14	0.09	0	0.06	0.23
LSCA (mm^2^)	Surface area	38.8	41.5	49.7	101	57	48
	SAR-TAWSS (%)	0	0	0	0	0	0
	SAR-OSI (%)	0	0	0	0	0	0
RSCA (mm^2^)	Surface area	31.4	35.8	61.2	111	93.7	43.9
	SAR-TAWSS (%)	0.8	0	0	1.3	0	0
	SAR-OSI (%)	0	0	0	0	0	0
LPCA (mm^2^)	Surface area	214	156	253	245	239	238
	SAR-TAWSS (%)	0	5.8	0.2	1.5	0.2	0.9
	SAR-OSI (%)	0	0	0	0.12	0	0
RPCA (mm^2^)	Surface area	249	155	266	204	249	213
	SAR-TAWSS (%)	0	0	0	0.4	0.1	0.9
	SAR-OSI (%)	0	0	0	0	0	0

**Fig 6:**
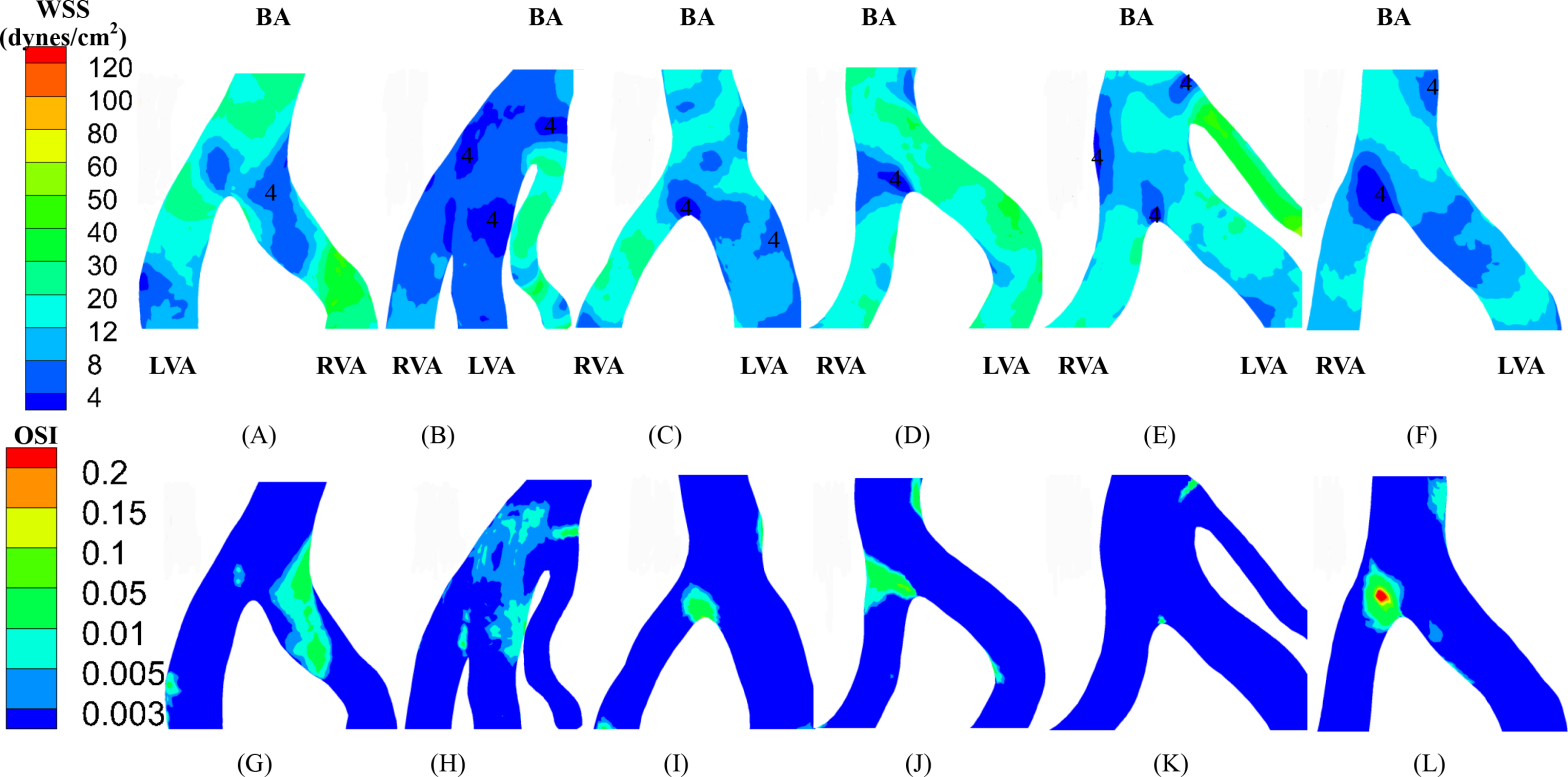
(A-F) TAWSS and (G-L) OSI near vertebrobasilar flow convergence with LVA and RVA merging into BA.

**Fig 7:**
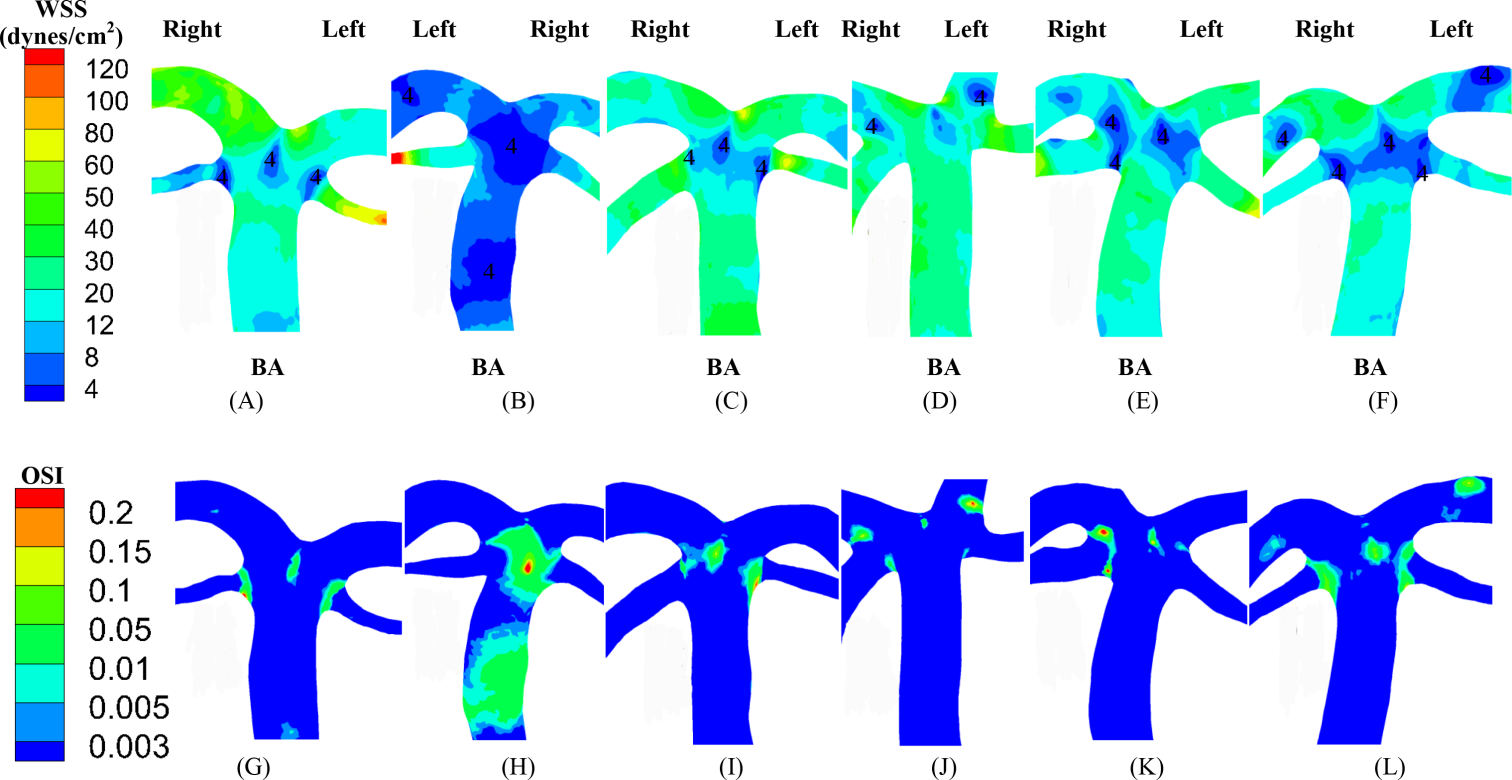
(A-F) TAWSS and (G-L) OSI near vertebrobasilar flow divergence with BA bifurcating into LSCA, RSCA, LPCA and RPCA.

The upstream flow convergence and downstream flow divergence are frequent sites for atherosclerosis and aneurysm, respectively. Low TAWSS and high OSI occurred near the two sites, as shown in Figs 6 and 7. Table 4 lists SAR-TAWSS and SAR-OSI in the two sites. Subject B had the maximal SAR-TAWSS (p value < 0.05) while subject D had the minimal SAR-TAWSS. Moreover, SAR-OSI had a low value (< 0.3%) and SAR-transWSS was zero in the two sites.

**Table 4:** Statistics for junctional surface area, SAR-TAWSS, and SAR-OSI at vertebrobasilar flow convergence and divergence.

Human subjects	A	B	C	D	E	F
Vertebrobasilar flow convergence with LVA and RVA merging into BA						
Junctional surface area (mm^2^)	330.57	379.83	524.41	409.56	460.91	452.01
SAR-TAWSS (%)	1.05	11.76	0.96	0.76	2.67	2.91
SAR-OSI (%)	0	0	0	0	0	0.14
Vertebrobasilar flow divergence with BA bifurcating into SCAs and PCAs						
Junctional surface area (mm^2^)	258.76	297.71	294.93	297.11	290.41	311.66
SAR-TAWSS (%)	4.96	21.46	2.85	1.11	3.11	5.74
SAR-OSI (%)	0.14	0.21	0.11	0.10	0.07	0.26

## Discussion

Based on 3D geometry and morphometry reconstructed from normal patient CTA images, we carried out a hemodynamic analysis in the vertebrobasilar arterial system that is comprised of LVA, RVA, BA, AICA, LSCA, RSCA, LPCA and RPCA. The blood was considered as a Newtonian fluid in the analysis given negligible non-Newtonian effects (Fig 2C vs. Figs 3A and 4A), which agreed with previous studies [24, 25]. Since Re_mean_ < 400 in Table 3, the blood flow was laminar in vertebrobasilar arteries of these normal patients albeit energetic, rapid fluctuations were observed in CFD models of bifurcation aneurysms [26]. The present study focused on the interplay of upstream flow confluence (LVA and RVA merging into BA) and downstream flow divergence (BA bifurcating into SCAs and PCAs) in the vertebrobasilar system. The corresponding findings were discussed below in turn.

Low TAWSS and high OSI (i.e., TAWSS ≤ 4 dynes/cm^2^ and OSI ≥ 0.15) are risk factors for the incidence of atherosclerosis and aneurysm [5, 6, 27, 28], which generally occur near curvatures, bifurcations, anastomoses and so on [21, 29–32]. At the flow confluence of vertebrobasilar system, Ravensbergen et al. showed atherosclerotic plaques at the anastomotic apex and lateral walls of the BA, which were associated with low TAWSS [9, 11]. They also found that a blunted apex and a large confluence angle were two geometric risk factors for the occurrence of an atherosclerotic plaque at the BA apex. Smith and Bellon demonstrated at least two flow patterns within the BA due to the non-admixture of vertebral artery flows [13]. Wake-Buck et al. presented the effects of curvatures and their orientations on the flow patterns [14]. In agreement with the conclusions in Refs. [9, 11, 13, 14], this study showed the distribution of low TAWSS and high OSI at the anastomotic apex and inner side of the curvature. It was, however, difficult to predict the incidence of atherosclerosis by simple geometrical zones (e.g., lateral walls, apex, the site opposite to the flow divider, etc.) given the complex hemodynamics at the upstream flow confluence. Alternatively, SAR-TAWSS, SAR-OSI, and SAR-transWSS were defined to quantify the hemodynamic environment, as shown in Table 4.

A key finding of the study is that SAR-OSI at the flow convergence was zero for subjects A-D (i.e., OSI values at all positions of the flow convergence were smaller than 0.15) and had a low value of 0.14% for subject F while SAR-transWSS was zero (i.e., transWSS values at all positions of the flow convergence were smaller than 6 dynes/cm^2^) for all patients. Since high OSI resulted from strong reversed flows [29], the flow reversal had small contributions to the incidence of atherosclerotic plaques at the flow convergence. Furthermore, as compared with the tuning fork geometry in subjects A-C, we found stronger spiral flows in the S-shaped or C-shaped walking geometry in subjects D-F (Fig 5A–5C vs. 5D–5F). The spiral flows initiated from the curvature of the dominant VA, which agreed with the computational results in Ref. [14]. The mixing of flows increased the swirling strength and led to the complex distribution of hemodynamic parameters at the flow convergence. The AICA functioned as a shunt path such that it weakened the secondary flows in the BA. Although the secondary flows were weakened at the flow divergence, they continued to propagate in the distal arteries. Mohamied et al. have shown that high transWSS could characterize the multidirectional flows [8]. Because of zero SAR-transWSS for all subjects, the secondary flows could have negligible effects on the incidence of atherosclerosis. It is known that low TAWSS coincided with stagnated, secondary, and reversed flows [29]. Hence, low TAWSS caused mainly by stagnated flows were a risk factor for the incidence of atherosclerosis at the flow convergence. On the other hand, the flow patterns near the flow divergence of vertebrobasilar system, similar to those at a junction in the cardiovascular system of other organs [21, 29–32], resulted in the non-regular distribution of hemodynamic parameters. Moreover, low TAWSS and high OSI occurred near the aneurysm-prone carina due to the oscillatory flows in the direction perpendicular to the BA centerline [31], which led to higher values of SAR-TAWSS and SAR-OSI at the downstream flow divergence than those at the upstream flow convergence.

Another key finding of the study is that the decrease in total outlet area (= ∑ outlet areas of SCAs and PCAs) could increase the vascular resistance to reduce the flow rate that significantly deteriorated the hemodynamic environment (i.e., a significant increase of SAR-TAWSS) in the vertebrobasilar system, as shown in Tables 3 and 4. Here, PCAs only included the P1 segment, which origins at the BA termination to the posterior communicating artery (PCOM), within interpeduncular cistern. Since a grey-scale threshold method with a low CT-threshold of 80 HU was selected to reconstruct the 3D geometry of vertebrobasilar system, this excluded small vessel segments distal to SCAs in the reconstruction. The artificial errors for determination of outlet surface areas were hence negligible. Subject B had the least total outlet area, but the highest SAR-TAWSS in each vessel and flow convergence and divergence of the vertebrobasilar system, as shown in Tables 3 and 4, as well as the lowest mean value of TAWSS. In contrast, subject D with the largest total outlet area had the smallest SAR-TAWSS. This illustrates the significant effects of cerebral microcirculation on the macrocirculation given the scaling relationship between the total outlet area and distal microvasculature [19].

### Implications for the posterior circulation

Subject B, the oldest patient, has the highest value of systolic blood pressure, pulse pressure (the difference between the systolic and diastolic pressures), cholesterol and triglycerides. These cardiovascular risk factors can impair the posterior microvasculature, which, in turn, exacerbates the hemodynamic environment in large arteries of vertebrobasilar system, as shown in Figs 3–7 and Tables 3 and 4. Although a previous study has shown that asymmetric VA flow could be a hemodynamic contributor of BA curvature and peri-vertebrobasilar junctional diseases [22], this study implies that the microcirculation injury should be a significant factor for the incidence and progression of atherosclerosis in large arteries of vertebrobasilar system, which requires further investigations.

### Critique of the model

The CFD simulation did not take vessel compliance into account because a previous study has shown negligible effects of vessel compliance on TAWSS and OSI [30]. Moreover, 3D reconstruction of vessels may be affected by imaging parameters and resolution. A study has recently shown that a low CT-threshold of 80 can satisfy the accuracy of 3D reconstruction [33]. The present study did not include human subjects with fPCA, i.e., a PCA arising from the internal carotid artery [34, 35], which needs to be considered in the following computational studies. Furthermore, future perspective studies in patients with stenoses and aneurysms should be carried out to validate predictions of the CFD simulation to the incidence and progression of atherosclerosis and aneurysm in posterior circulation.

## Conclusions

This retrospective study performed a hemodynamic analysis in patient vertebrobasilar system. The interplay of upstream flow confluence and downstream flow divergence was found to significantly affect the distribution of hemodynamic parameters (e.g., streamlines, TAWSS, OSI and transWSS) in patients of no stenoses and aneurysms. The outlet resistance resulting from the distal microvasculature should be accurately estimated when a CFD simulation is carried out in large arteries of vertebrobasilar system. This study provides insight for understanding of the posterior circulation relevant to the potential incidence of atherosclerosis and aneurysms.

## Appendix

### Resistance boundary condition

The total resistance at the inlet of LVA and RVA, *R*_*total*,_ is determined as:

$$R_{total}=\left(P-P_{0}\right)/Q_{total}$$ [A1]

$$Q_{total}=\overset{\text{RVA}}{\underset{\text{LVA}}{\sum }}{\text{v}}_{inlet}\times \frac{\pi }{4}\times D_{inlet}^{2}={\text{v}}_{BA}\times \frac{\pi }{4}\times D_{BA}^{2}$$ [A2]

where v_*inlet*_ and *D*_*inlet*_ refer to the flow velocity and diameter at the inlet of LVA and RVA; v_*BA*_ and *D*_*BA*_ refer to the flow velocity and diameter in BA. The total cerebral flow, *Q*_*total*_, is estimated from CT images of cerebral mass using the scaling law [36, 37]. The zero-flow pressure, *P*_0_, is set to 35 mmHg [38].

Since the diameter-flow scaling law occurs in the cerebral circulation [20] similar to the coronary circulation [19, 36, 39], the resistance at each outlet of the vertebrobasilar system, *R*_*outlet*_, can be written as:

$$R_{outlet}=R_{total}\times {\left(\frac{D_{BA}}{D_{outlet}}\right)}^{7/3}=\frac{P_{outlet}-P_{0}}{{\text{v}}_{outlet}\times \frac{\pi }{4}\times D_{outlet}^{2}}$$ [A3]

where *D*_*outlet*_ refers to the diameter at each outlet of the vertebrobasilar system. Hence, we can get the following equation as:

$${\text{v}}_{outlet}=\frac{P_{outlet}-P_{0}}{R_{outlet}\times \frac{\pi }{4}\times D_{outlet}^{2}}=\frac{P_{outlet}-P_{0}}{R_{total}\times {\left(\frac{D_{BA}}{D_{outlet}}\right)}^{7/3}\times \frac{\pi }{4}\times D_{outlet}^{2}}$$ [A4]

where *P*_*outlet*_ refers to the pressure at each outlet of the vertebrobasilar system. Eq [A4] was used as the resistance boundary condition with v_*outlet*_ and *P*_*outlet*_ being the variables.

### Hemodynamic parameters

Similar to a previous study [18], at any point of 3-D meshes, the stress can be represented as a nine-component tensor ($\bar{\bar{\tau }}$), which can be written as follows:

$$\bar{\bar{\tau }}=\left[\begin{array}{ccc} \tau_{11} & \tau_{12} & \tau_{13}\\ \tau_{21} & \tau_{22} & \tau_{23}\\ \tau_{31} & \tau_{32} & \tau_{33} \end{array}\right]=2\mu \bar{\bar{D}}=\mu \left[\begin{array}{ccc} 2\frac{\partial u}{\partial x} & \frac{\partial u}{\partial y}+\frac{\partial v}{\partial x} & \frac{\partial u}{\partial z}+\frac{\partial w}{\partial x}\\ \frac{\partial u}{\partial y}+\frac{\partial v}{\partial x} & 2\frac{\partial v}{\partial y} & \frac{\partial v}{\partial z}+\frac{\partial w}{\partial y}\\ \frac{\partial u}{\partial z}+\frac{\partial w}{\partial x} & \frac{\partial v}{\partial z}+\frac{\partial w}{\partial y} & 2\frac{\partial w}{\partial z} \end{array}\right]$$ [A5]

where $\bar{\bar{D}}$ is the shear rate tensor. The stress on the wall, its normal component, and its two tangential components can be written as, respectively:

$$\vec{\tau }=\bar{\bar{\tau }}\cdot \text{n},\;\tau_{n}=\text{n}\cdot \bar{\bar{\tau }}\cdot \text{n},\;\tau_{t_{1}}={\text{t}}_{1}\cdot \bar{\bar{\tau }}\cdot \text{n}\;\text{and}\;\tau_{t_{2}}={\text{t}}_{2}\cdot \bar{\bar{\tau }}\cdot \text{n}$$ [A6]

where n, t_1_, and t_2_ are the unit vector in the normal and two tangential directions, respectively. The shear component of $\vec{\tau }$ has the vector form:

$${\vec{\tau }}_{\text{shear}}=\vec{\tau }-\left(\vec{\tau }\cdot \text{n}\right)\text{n}$$ [A7]

Eq [A7] is used to calculate WSS, which has a magnitude: $\left|{\vec{\tau }}_{\text{shear}}\right|=\sqrt{\vec{\tau }\cdot \vec{\tau }-{\left(\vec{\tau }\cdot \text{n}\right)}^{2}}$. The TAWSS can be written as follows:

$$\text{TAWSS}=\frac{1}{T}\int_{0}^{T}\left|{\vec{\tau }}_{\text{shear}}\right|\cdot dt$$ [A8]

The OSI can be written as follows:

$$\text{OSI}=\frac{1}{2}\left[1-\frac{\left|\frac{1}{T}\int_{0}^{T}{\vec{\tau }}_{\text{shear}}\cdot dt\right|}{\frac{1}{T}\int_{0}^{T}\left|{\vec{\tau }}_{\text{shear}}\right|\cdot dt}\right]$$ [A9]

The transWSS can be written as follows:

$$\text{transWSS}=\frac{1}{T}\int_{0}^{T}\left|{\vec{\tau }}_{\text{shear}}\cdot \left[\text{n}\times \frac{\int_{0}^{T}{\vec{\tau }}_{\text{shear}}\cdot dt}{\left|\int_{0}^{T}{\vec{\tau }}_{\text{shear}}\cdot dt\right|}\right]\right|\cdot dt$$ [A10]

Eqs [A8–A10] were used to calculate the TAWSS, OSI, and transWSS, respectively.
